# Southern range extension of Spix's saddle-back tamarin, *Leontocebus fuscicollis fuscicollis*, in Peru

**DOI:** 10.5194/pb-9-19-2022

**Published:** 2022-07-22

**Authors:** Elvis Charpentier, Gabriel García-Mendoza, José Cruz-Guimaraes, Rolando Aquino, Eckhard W. Heymann

**Affiliations:** 1 Equipo Primatológico del Perú (EPP), Calle Internacional M-43, Iquitos, Peru; 2 Verhaltensökologie & Soziobiologie, Deutsches Primatenzentrum, Leibniz-Institut für Primatenforschung, Kellnerweg 4, 37077 Göttingen, Germany

## Abstract

Peru has the highest diversity of members of the tamarin genus *Leontocebus* (Callitrichidae). However, for a number of taxa from this genus the distributional ranges are still not well known. In this paper we provide evidence for the extension of the southern range of *Leontocebus fuscicollis fuscicollis* to the right bank of the Río Abujao, south of which it is replaced by *Leontocebus weddelli weddelli*.

## 1 Introduction

The genus *Leontocebus* comprises the tamarin taxa formerly of the genus *Saguinus* that were named by Hershkovitz (1977) as the *nigricollis* group of white-mouth tamarins (Rylands et al., 2016). It is widely distributed in western Amazonia, i.e. eastern Ecuador, southern Colombia, eastern Peru, northern Bolivia, and western Brazil (Rylands and Mittermeier, 2013; Rylands et al., 2016). In the west, its range is restricted by the Andean Cordillera; in the north by the rivers Caquetá, Caguán and Orteguaza; and in the east by the rivers Purús and Ji-Paraná. In Bolivia it reaches to about 16 ${}^{\circ }$ S with no clearly defined geographic boundary. The ranges of the species and subspecies are generally separated by major rivers (Hershkovitz, 1977), although river barriers may break down at the headwaters, as is the case for *Leontocebus fuscicollis fuscicollis * and *Leontocebus weddelli melanoleucus*
We refer the readers to the table in the Supplement for currently accepted and previously used names of species and subspecies.
(Peres et al., 1996), or through the transfer of a population from one side of the river to the other side due to lateral migration of river channels or meander cutoffs, as in the case of *Leontocebus illigeri* (see Hodun et al., 1981).

**Figure 1:**
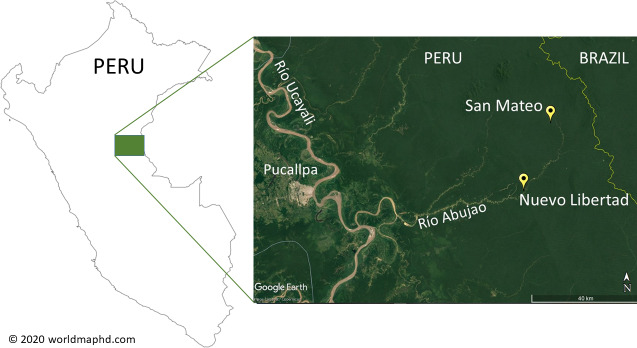
Location of the survey areas in Peru. Country map © World Map HD 2020 (https://www.worldmaphd.com/, last access: 30 May 2022); satellite image © Google Earth 2022.

A total of 10 of the currently recognized 17 taxa of *Leontocebus* are found in Peru (Aquino et al., 2015; Rylands et al., 2016), amongst them Spix's saddle-back tamarin, *L. f. fuscicollis*. This is one of three subspecies of *L. fuscicollis*, the others being *Leontocebus fuscicollis avilapiresi * and *Leontocebus fuscicollis primitivus* (see Rylands et al., 2016). They are likely to be elevated to species rank once they are included in genetic studies. In Peru, the presence of *L. f. fuscicollis* was first reported by Soini (1990). Its range is restricted in the north by the Río Blanco, a right bank tributary of the Río Tapiche in the Department of Loreto, and in the west by the Río Tapiche (Hershkovitz, 1977; Aquino and Encarnación, 1994). However, its southern limit has not been clearly defined. In Map 2 in Aquino and Encarnación (1994), *Leontocebus nigrifrons* is found both north and south of the range of *L. f. fuscicollis*. However, this is highly unlikely, as the range of the latter extends into Brazil (unlike *L. nigrifrons* which is endemic to Peru, restricted by the Río Yavarí in the east) and thus would disrupt the distribution of the former. Further to the south, in the Department of Ucayali, the Río Abujao is the northern limit of *Leontocebus weddelli weddelli* (Aquino and Encarnación, 1994; Aquino et al., 2015). It is thus conceivable that the range of *L. f. fuscicollis* would extend south to the north bank of this river. Here we provide observational and photographic evidence for the presence of *L. f. fuscicollis* as far south of its previously known range as the Río Abujao.

## 2 Methods

In 2015 and 2018, Elvis Charpentier, Gabriel García-Mendoza, and José Cruz-Guimaraes conducted mammal inventories in two areas north of the Río Abujao, around the indigenous communities (Comunidades Nativas) of San Mateo (73 ${}^{\circ }$ $43.1^{\prime }$ S, 8 ${}^{\circ }$ $9.5^{\prime }$ W; easting 641197, northing 9098028; UTM 18L) and Nuevo Libertad ( 73 ${}^{\circ }$ $49.5^{\prime }$ S, 8 ${}^{\circ }$ $22.9^{\prime }$ W; easting 629437, northing 9073340; Fig. 1), in the framework of the project “Propuesta para el Establecimiento del Area de Conservación Regional de la Cuenca Alta del Río Tamaya-Abujao” (Proposal for the Establishment of the Regional Conservation Area in the Upper Reaches of the River Tamaya-Abujao).

## 3 Results and discussion

**Figure 2:**
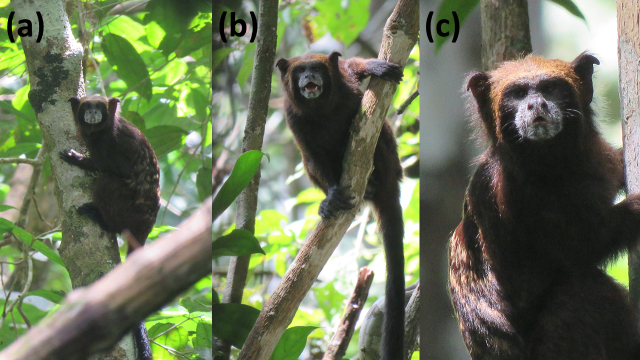
Adult *Leontocebus fuscicollis fuscicollis*. Note the brown forehead and the light brown line above the eyebrows and at the sideburns that are typical for this taxon. Panels **(a)** and **(b)** show the same individual (photos: Elvis Charpentier). **(c)** Photo: Gabriel García-Mendoza.

During the mammal inventories we observed and photographed (Gabriel García-Mendoza on 27 June 2015 and Elvis Charpentier on 12 March 2018, respectively), tamarins that phenotypically match *L. f. fuscicollis* (Fig. 2). The orange-brownish forehead, crown and temples distinguish them unequivocally from all other *Leontocebus* taxa that could potentially occur in the region: the black forehead and crown in *L. illigeri* and *L. leucogenys*, the black forehead in *L. nigrifrons*, and the white frontal blaze in *L. w. weddelli* (Hershkovitz, 1977; Groves, 2001; Rylands et al., 2016). Altogether, we have seen *L. f. fuscicollis* on nine occasions (eight times at San Mateo, once at Nuevo Libertad). The sightings were in terra firme forest (“bosque de altura”; Encarnación 1985). *Leontocebus f. fuscicollis* is sympatric with *Saguinus m. mystax*, the distribution of which extends further south to the Río Inuya (Hershkovitz, 1977; Heymann et al., 2018), but we have not seen the two tamarin species in mixed-species groups that are common in other areas of sympatry between two tamarin species (Heymann and Buchanan-Smith, 2000).

**Figure 3:**
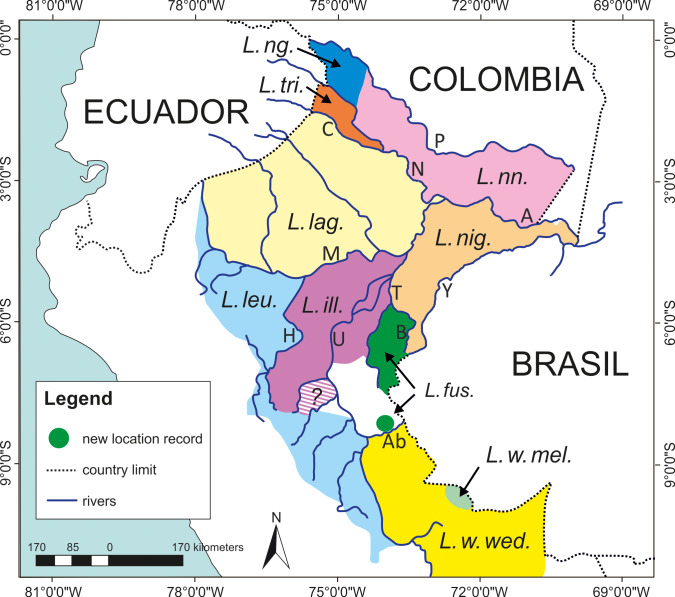
Geographic distribution of *Leontocebus* taxa in Peru and the new location record for *Leontocebus* *fuscicollis* (green dot). *L.fus.* – *Leontocebus* *fuscicollis* *fuscicollis*; *L.ill.* – *Leontocebus illigeri*; *L.lag.* – *Leontocebus lagonotus*; *L.leu.* – *Leontocebus leucogenys*; *L.ng.* – *Leontocebus nigricollis graellsi*; *L.nig.* – *Leontocebus nigrifrons*; *L.nn.* – *Leontocebus nigricollis nigricollis*; *L.tri.* – *Leontocebus tripartitus*; *L.w.mel.* – *Leontocebus weddelli melanoleucus*; *L.w.wed.* – *Leontocebus weddelli weddelli*. Letters denote the names of rivers: A – Río Amazonas; Ab – Río Abujao; B – Río Blanco; C – Río Curaray; H – Río Huallaga; M – Río Marañón; P – Río Putumayo; T – Río Tapiche; U – Río Ucayali; Y – Río Yavarí. Note that the geographic distribution of *L. f. fuscicollis*, *L. w. melanoleucus*, and *L. w. weddelli* extend into Brazil.

Our records extend the southern range of *L. f. fuscicollis*, very likely making the Río Abujao its southern limit, south of which it is replaced by *L. w. weddelli* (Fig. 3). However, at the headwaters of the Río Abujao the two species could come into contact. Similarly, at the headwaters of the Río Tapiche, *L. f. fuscicollis* could come into contact with *L. illigeri* but the southern limit to the range of *L. illigeri* is not well defined (Aquino and Encarnación, 1994). This raises the question whether hybridization may take place there, as observed between * L. f. fuscicollis* and *L. w. melanoleucus* in Brazil (Peres et al., 1996), or possible introgression resulting in incongruity between genetic and phenotypic data, as in *Leontocebus leucogenys* and *L. illigeri* (see Matauschek et al., 2011). Genetic analyses will be needed to clarify this issue. Also, some individuals could be trapped and photographed, to document in more detail the phenotypic characteristics. The range extension adds to another extension, namely that of *L. w. melanoleucus* (see Mena et al., 2007), and illustrates that we still lack a complete picture of the distribution of tamarins (and also of other Amazonian primates), particularly from areas that have not been explored in the past, either because they are difficult to access or because cocaine production makes surveys in some areas too risky. Unfortunately, the recent increase in deforestation rates in Amazonia leaves little hope that we might ever be able to obtain a more comprehensive picture.

## Supplement

10.5194/pb-9-19-2022-supplement The supplement related to this article is available online at: https://doi.org/10.5194/pb-9-19-2022-supplement.

## Data Availability

No data sets were used in this article.

## References

[bib1.bib1] Aquino R, Encarnación F (1994). Primates of Peru – Los Primates del Perú. Primate Rep.

[bib1.bib2] Aquino R, Cornejo FM, Cortés Ortiz L, Encarnación CF, Heymann EW, Marsh LK, Mittermeier RA, Rylands AB, Vermeer J (2015). Monkeys of Peru. Pocket identification guide, Tropical Pocket Guide Series.

[bib1.bib3] Encarnación F (1985). Introducción a la flora y vegetación de la Amazonía peruana: estado actual de los estudios, medio natural y ensayo de una clave de determinación de las formaciones vegetales en la llanura amazónica. Candollea.

[bib1.bib4] Groves C (2001). Primate taxonomy.

[bib1.bib5] Hershkovitz P (1977). Living New World monkeys (Platyrrhini), vol 1.

[bib1.bib6] Heymann EW, Buchanan-Smith HM (2000). The behavioural ecology of mixed-species troops of callitrichine primates. Biol Rev.

[bib1.bib7] Heymann EW, Mittermeier RA, Rylands AB (2018). The IUCN Red List of Threatened Species, e.T41526A17932444.

[bib1.bib8] Hodun A, Snowdon CT, Soini P (1981). Subspecific variation in the long calls of the tamarin, *Saguinus fuscicollis*. Z Tierpsychol.

[bib1.bib9] Matauschek C, Roos C, Heymann EW (2011). Mitochondrial phylogeny of tamarins (*Saguinus*, Hoffmannsegg 1807) with taxonomic and biogeographic implications for the *S. nigricollis* species group. Am J Phys Anthropol.

[bib1.bib10] Mena JL, Dosantos A, Grocio Gil J, Escobedo M, Aquino R, Peres J (2007). Primer registro de *Saguinus fuscicollis melanoleucus* (Miranda Ribeiro, 1912) en la Amazonia peruana. Rev peru Biol.

[bib1.bib11] Peres CA, Patton JL, da Silva MNF (1996). Riverine barriers and gene flow in Amazonian saddle-back tamarins. Folia Primatol.

[bib1.bib12] Rylands AB, Mittermeier RA, Mittermeier RA, Rylands AB, Wilson DE (2013). Handbook of the mammals of the world. 3. Primates.

[bib1.bib13] Rylands AB, Heymann EW, Lynch Alfaro J, Buckner JC, Roos C, Matauschek C, Boubli JP, Sampaio R, Mittermeier RA (2016). Taxonomic review of the New World tamarins (Primates: Callitrichidae). Zool J Linn Soc.

[bib1.bib14] Soini P, Castro-Rodríguez NE (1990). La primatología en el Perú. Investigaciones primatológicas (1973–1985).

