# Supplementary material for: Southern range extension of Spix's saddle-back tamarin, Leontocebus fuscicollis fuscicollis, in Peru
Source: Primate Biol. 2022 Jul 22;9(2):19–22. doi: 10.5194/pb-9-19-2022 (PMC9399907; doi:10.5194/pb-9-19-2022)
Supplement: The supplement related to this article is available online at: https://doi.org/10.5194/pb-9-19-2022-supplement. [file pb-9-19-supplement.pdf]

Supplement of Primate Biol., 9, 19–22, 2022
https://doi.org/10.5194/pb-9-19-2022-supplement
© Author(s) 2022. CC BY 4.0 License.

Primate Biology
Open Access

Supplement of

Southern range extension of Spix's saddle-back tamarin, *Leontocebus fuscicollis fuscicollis*, in Peru

Elvis Charpentier et al.

Correspondence to: Eckhard W. Heymann (eheyman@gwdg.de)

The copyright of individual parts of the supplement might differ from the article licence.

Supplementary Table.

Currently recognized taxon names (Rylands et al., 2016) and names used previously in publications quoted in Charpentier et al.

Current names	Previous names	References for previous names
Leontocebus fuscicollis	*Saguinus fuscicollis*	Hershkovitz 1977; Aquino & Encarnacion 1994; Groves 2001
Leontocebus fuscicollis avilapiresi	*Saguinus fuscicollis avilapiresi*	Hershkovitz 1977; Peres et al. 1996; Groves 2001
Leontocebus fuscicollis fuscicollis	*Saguinus fuscicollis fuscicollis*	Hershkovitz 1977; Aquino & Encarnacion 1994; Peres et al. 1996; Groves 2001
Leontocebus fuscicollis primitivus	*Saguinus fuscicollis primitivus*	Hershkovitz 1977; Peres et al. 1996; Groves 2001
Leontocebus illigeri	*Saguinus fuscicollis illigeri*	Hershkovitz 1977; Hodun et al. 1981; Aquino & Encarnacion 1994; Groves 2001
	Saguinus illigeri	Matauschek et al. 2011
Leontocebus lagonotus	*Saguinus fuscicollis lagonotus*	Hershkovitz 1977; Aquino & Encarnacion 1994, Groves 2001
	Saguinus lagonotus	Matauschek et al. 2011
Leontocebus leucogenys	*Saguinus fuscicollis leucogenys*	Hershkovitz 1977; Aquino & Encarnacion 1994; Groves 2001
	Saguinus leucogenys	Matauschek et al. 2011
Leontocebus nigricollis graellsi	*Saguinus nigricollis graellsi*	Hershkovitz 1977; Aquino & Encarnacion 1994; Matauschek et al. 2011
	Saguinus graellsi	Groves 2001

Leontocebus nigricollis nigricollis	*Saguinus nigricollis nigricollis*	Hershkovitz 1977; Aquino & Encarnacion 1994; Matauschek et al. 2011
Leontocebus nigrifrons	*Saguinus fuscicollis nigrifrons*	Hershkovitz 1977; Aquino & Encarnacion 1994
	Saguinus nigrifrons	Matauschek et al. 2011
Leontocebus tripartitus	*Saguinus fuscicollis tripartitus*	Hershkovitz 1977
	Saguinus tripartitus	Thorington 1988; Aquino & Encarnacion 1994; Matauschek et al. 2011
Leontocebus weddelli melanoleucus	*Saguinus fuscicollis melanoleucus*	Hershkovitz 1977; Peres et al. 1996; Mena et al. 2007
	Saguinus melanoleucus	Groves 2001
Leontocebus weddelli weddelli	*Saguinus fuscicollis weddelli*	Hershkovitz 1977; Peres et al. 1996; Groves 2001; Mena et al. 2007

References

Aquino R, Encarnación F (1994) Primates of Peru - Los Primates del Perú. Primate Report 40:1-127

Groves C (2001) Primate taxonomy. Smithsonian Institution Press, Washington

Hershkovitz P (1977) Living New World monkeys (Platyrrhini), vol. 1. University of Chicago Press, Chicago

Hodun A, Snowdon CT, Soini P (1981) Subspecific variation in the long calls of the tamarin, *Saguinus fuscicollis*. Zeitschrift für Tierpsychologie 57:97-110

Matauschek C, Roos C, Heymann EW (2011) Mitochondrial phylogeny of tamarins (*Saguinus*, Hoffmannsegg 1807) with taxonomic and biogeographic implications for the *S. nigricollis* species group. American Journal of Physical Anthropology 144:564-574

Mena JL, Dosantos A, Grocio Gil J, Escobedo M, Aquino R, Peres J (2007) Primer registro de *Saguinus fuscicollis melanoleucus* (Miranda Ribeiro, 1912) en la Amazonia peruana. Revista Peruana de Biología 14:39-42

Peres CA, Patton JL, da Silva MNF (1996) Riverine barriers and gene flow in Amazonian saddle-back tamarins. Folia Primatologica 67:113-124

Thorington RW, Jr (1988) Taxonomic status of *Saguinus tripartitus* (Milne-Edwards, 1878). American Journal of Primatology 15:367-371
